# Validation of smartphone inclinometer tools for measuring rib hump in scoliosis patients

**DOI:** 10.1186/1748-7161-10-S1-O28

**Published:** 2015-01-19

**Authors:** Angela Guardia, Muhammad Imran Khan, Andreas Donauer, Kajsa Duke

**Affiliations:** 1 Department of Mechanical Engineering, University of Alberta, Canada; 2 Certified Orthotist, Glenrose Rehabilitation Hospital, Alberta Health Services, Canada; 3 Division of Orthopaedic Surgery, University of Alberta, Canada

## Objective

To validate smartphone inclinometer tools to measure rib hump in scoliosis patients.

## Material and methods

**Figure 1:**
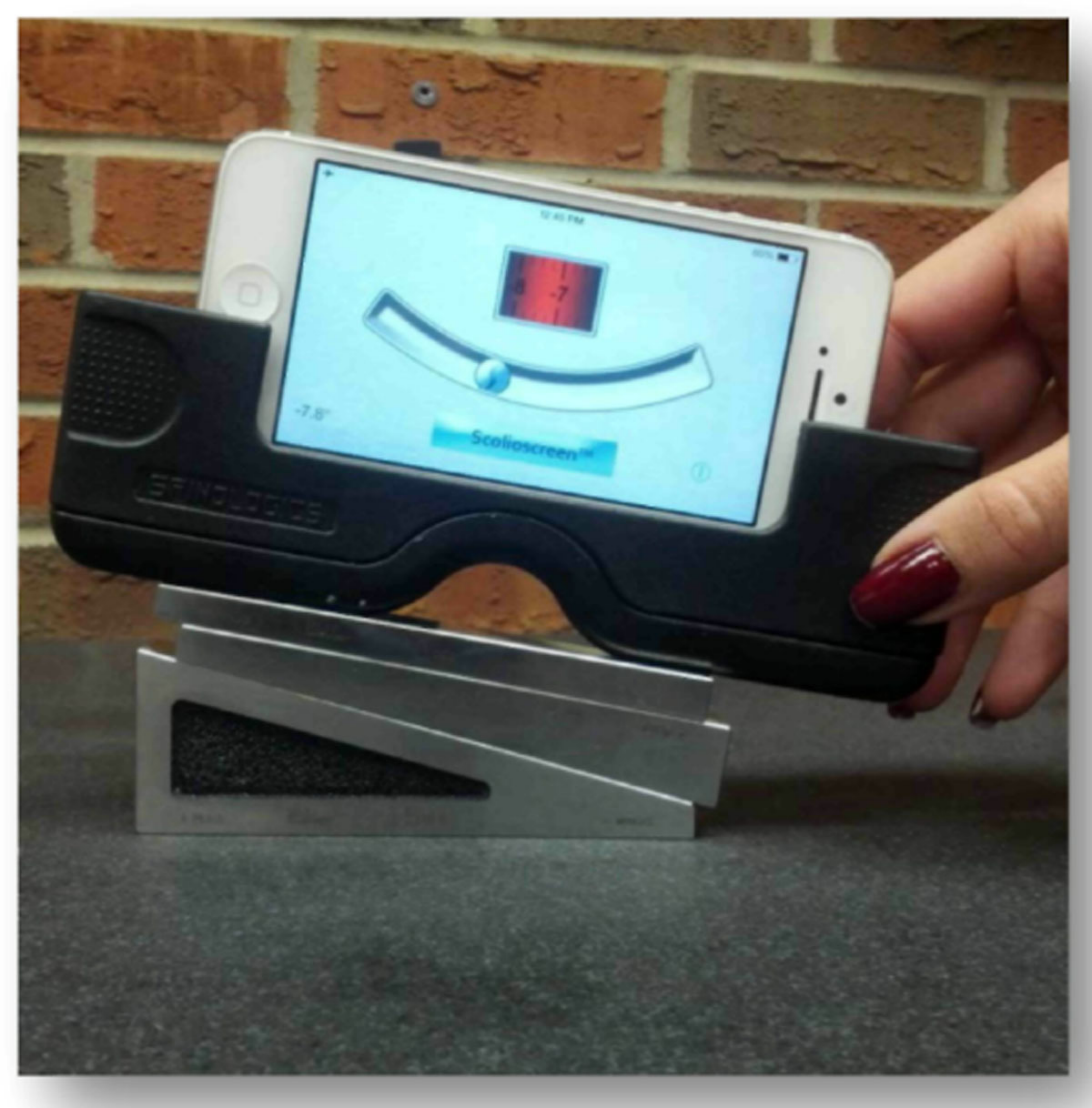
The iPhone application called Scolioscreen along with a rubber sleeve placed on the angle gauge blocks.

Inclinometer tools in smartphones can be used to measure angles. Spinologics (Montreal, Canada), designed an iPhone application called Scolioscreen along with a rubber sleeve that can be placed on the iPhone to take rib hump measurements. To ensure the new device’s ability to measure angles, measurements were taken on a flat granite block which was accurately leveled in the Department of Mechanical Engineering Metrology Lab. Highly accurate angle gage blocks were used (0.0003 degrees). Angles from -30 to 30 degrees were measured by two observers, with two different iPhones (iPhone4 and iPhone5), on two occasions. Angles blocks were arranged by one observer at a random angle between -30 and 30 while the other blindly measured (Figure 1). Additionally, four plaster casts from brace patients were measured with one iPhone.

## Results

**Figure 2:**
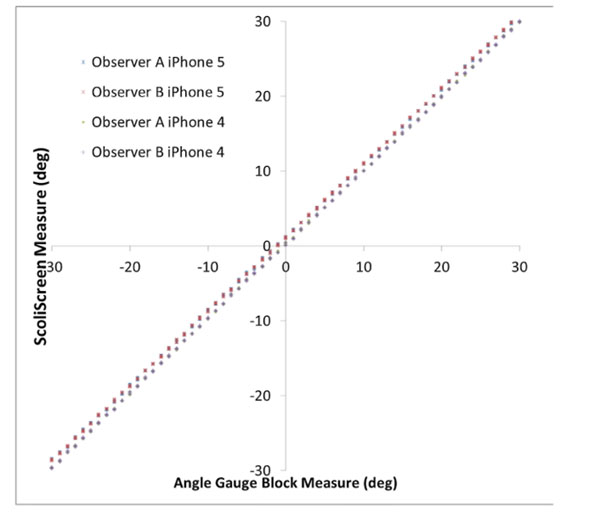
ScoliScreen measured value versus angle gauge block measures.

**Figure 3:**
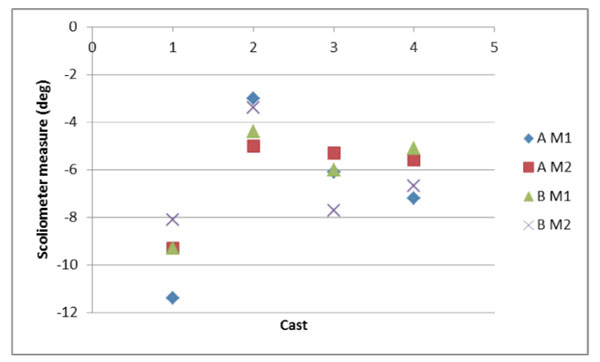
Variability in cast measures.

A total of 448 measures were made using angle blocks and 16 on the casts. There were no differences found between the two observers or the two different occasions on the angle blocks (Figure 2). One iPhone, however, consistently measured incorrectly by an average of -1.1 degrees (-0.7 to -1.6). Measures on the four casts ranged from 4 to 9.5 degrees. The variability of measuring the casts was greater with a maximum difference of 3.3 degrees (Figure 3).

## Conclusions

When compared to accurate angle blocks the iPhone Scoliscreen takes consistent measures. Some improvements can be made to the application. Namely, the addition of a calibration or zeroing feature. Caution should be used if different phones are used to take measures or if the iPhone is tilted. As expected, measuring the casts was less repeatable than the precise angle gauge blocks. However, it is safe to conclude that this application could be used to measure the trunk deformation on scoliotic patients.

